# A Profile of Injuries Sustained by Law Enforcement Officers: A Critical Review

**DOI:** 10.3390/ijerph14020142

**Published:** 2017-02-03

**Authors:** Kate Lyons, Cameron Radburn, Robin Orr, Rodney Pope

**Affiliations:** 1 Bond Institute of Health and Sport, Bond University, Gold Coast QLD 4229, Australia; kate.lyons@student.bond.edu.au (K.L.); cameron.radburn@student.bond.edu.au (C.R.); rpope@bond.edu.au (R.P.); 2 Tactical Research Unit, Bond University, Gold Coast QLD 4229, Australia

**Keywords:** police, law enforcement, injury, tactical, occupational health

## Abstract

Due to the unpredictable, varied and often physical nature of law enforcement duties, police officers are at a high risk of work-related physical injury. The aim of this critical narrative review was to identify and synthesize key findings of studies that have investigated musculoskeletal injuries sustained by law enforcement officers during occupational tasks. A systematic search of four databases using key search terms was conducted to identify potentially relevant studies, which were assessed against key inclusion and exclusion criteria to determine studies to be included in the review. Included studies were critically appraised and the level of evidence determined. Relevant data were extracted, tabulated and synthesized. The 16 identified studies ranged in percentage quality scores from 25.00% to 65.00%, with a mean score of 41.25% and high interrater agreement in scores reflected in a Cohen’s Kappa coefficient, κ = 0.977. The most common body site of injury was the upper extremity, the most common injury types were soft-tissue sprains and strains and the most common cause of injury was a non-compliant offender, often involving assault. However, there was limited peer reviewed research in this area and the published research had a narrow focus and was of low to fair methodological quality.

## 1. Introduction

Due to the unpredictable, varied, and physical nature of law enforcement, police officers are at a high risk of work-related physical injury [1]. The physical demands of law enforcement duties may include running varied distances with and without loads, restraining non-compliant offenders, carrying injured or unconscious people, self-defence manoeuvres, and manual handling tasks [2]. Sudden external forces when controlling a suspect or offender who is resisting arrest, trips and falls, and strains and sprains can all contribute to the high risk of injury within the law enforcement occupation [3].

As a result of these factors, law enforcement officers are at a greater risk of physical injury, specifically musculoskeletal injuries, than employees in many other occupations [3]. This greater injury risk has an impact not only on the worker, but also on the organization, which has to bear both the financial and workforce burdens of days lost due to injury and the post-injury rehabilitation or workers’ compensation costs [4,5,6,7]. These costs vary greatly within the literature, depending on the severity of injury, the length of time off work and the country in which the study was completed. A recent study found that the estimated cost associated with an injured law enforcement employee varied from $2500 to $12,000 (USD), depending on the area injured [5]. Previous studies have speculated that changes to post-injury rehabilitation may be needed in an attempt to reduce the duration of time that law enforcement officers are off work and the associated financial burden [6].

By determining the current profile of sites, mechanisms and types of injuries sustained by law enforcement officers, informed rehabilitation practices and injury prevention strategies for officers can be developed. For example, by knowing the mechanism of an injury individualised programs to increase post injury resilience to these mechanism can be developed, which will enable better control of gradual loading and return to work conditioning. In addition, by knowing the mechanism of an injury, targeted injury prevention strategies, like pre-conditioning (e.g., strengthening exercises) [8] or other reduction methods (e.g., changing a floor surface) [9] can be applied. These approaches will in turn may have an impact on injury incidence and reducing injury severity [10]. Therefore, the aim of this critical narrative review was to identify, critically appraise and synthesize key findings from recent literature investigating musculoskeletal injuries in the law enforcement profession, in order to develop a profile of the injuries experienced by this unique population.

## 2. Methods

A two-tiered approach was used to identify studies of relevance to this review. First, a systematic search of key databases, using carefully-selected search terms, was completed on 16 September 2016. Search terms were identified through completing a rapid literature review testing different key words and examining the terms used in known research in this area. Once key words were identified, authors (K.L., C.R.) used the databases to compile the search phrases listed in Table 1.

Secondly, additional studies of relevance from both peer-reviewed full-text sources and grey literature were sourced from known researchers in this field. Following removal of duplicates, all identified studies were then screened by title and abstract using pre-defined inclusion and exclusion criteria. Injury definitions varied across 5 of the included studies [6,11,12,13,14]. In the remaining studies injury was not defined. Considering this, for the purpose of this review, all studies which investigated law enforcement officers who were considered to have sustained ‘an injury’ were included. Several studies [7,13,14,15,16,17] did not specify whether the reported injuries occurred while the officer was on duty, whereas the remaining studies only included injuries that occurred whilst an officer was on duty [5,6,11,12,15,18,19,20,21,22]. The full criteria are detailed in Table 2. During the screening process, any studies which indicated potential to meet the eligibility criteria were obtained in full-text and reviewed in detail to determine final eligibility using the selection criteria. 

To limit bias and to ensure objective selection of studies at the screening and full text selection stages, two reviewers separately screened and selected studies using the inclusion and exclusion criteria (K.L., C.R.). Any disagreements regarding which studies should move to the next stage were resolved by discussion and consensus.

Through this approach, search bias, duplication bias, inclusion criteria bias, and selector bias [23] were limited. The search terms were kept broad to be more inclusive of studies and to limit search bias; duplicates were removed as the first line of screening to limit duplication bias; and finally, to limit selector bias, two reviewers screened the abstracts and then selected full text studies for inclusion in the review. The inclusion and exclusion criteria were established prior to screening commencing. 

The included studies were critically appraised using a modified Downs and Black checklist. This checklist is designed to assess the methodological quality and provide a profile of study strengths and weaknesses for both randomized control trials and non-randomized studies [24,25]. This checklist utilizes twenty-seven questions to assess the methodological quality of studies and has been used in reviews involving tactical populations [24,26,27,28,29]. A modified version of this tool was used, with question 27, relating to statistical power, modified to award one point for a ‘yes’ answer, indicating the authors had reported a sample size or power analysis, or zero points for a ‘no’ answer, indicating they had not. This revised scoring approach for question 27 replaced the original 6-point scoring scale, ranging from zero to five points, and reduced the maximum possible raw score for the Downs and Black checklist to 28, from the original 32 points [24]. This approach has been used previously in research due to subjectivity in the interpretation of the original question [25]. In addition to this modification, for the purposes of assessing methodological quality of studies included in the current review, several additional questions were ignored in the Down’s and Black checklist (Q’s 4, 8, 13, 14, 15, 19, 23 & 24) [24], as they were designed to evaluate aspects of methodological quality that were specific to experimental studies rather than the epidemiological, observational research designs of the included studies. For example, checklist question 14 asks ‘Was an attempt made to blind study subjects to the intervention they have received?’. The use of this additional modification to the Downs and Black checklist was not considered to have impacted on the analysis of study quality and meant that the included studies were not assessed against criteria for which they were never designed. This modification adjusted the maximum possible total raw score for the Downs and Black checklist to 20 points.

The critical appraisal of the included studies was completed individually by two authors (K.L., C.R.), with the level of interrater agreement on assigned scores then determined by a third author (R.O.) through calculation of a Cohen’s kappa coefficient (κ). This level of agreement was graded qualitatively, using previously published guidelines [30]. Following the Kappa analysis, any discrepancies in scores between the two primary raters were adjudicated by the third author (R.O.) to finalise the Critical Appraisal Scores (CAS). The qualitative ratings of methodological quality proposed by Kennelly [31] for specific ranges of total scores on the Downs and Black checklist were used to grade the studies. Due to the modifications made to the Downs and Black checklist, discussed above, all scores were first converted to percentages to enable grading using the approach of Kennelly [31]. On this basis, the grading criteria applied when rating the methodological quality of the included studies were as follows: Downs and Black total score <45.4%, ‘poor’ methodological quality; 45.4%–61.0%, ‘fair’ methodological quality; and >61.0%, ‘good’ methodological quality.

**Figure 1:**
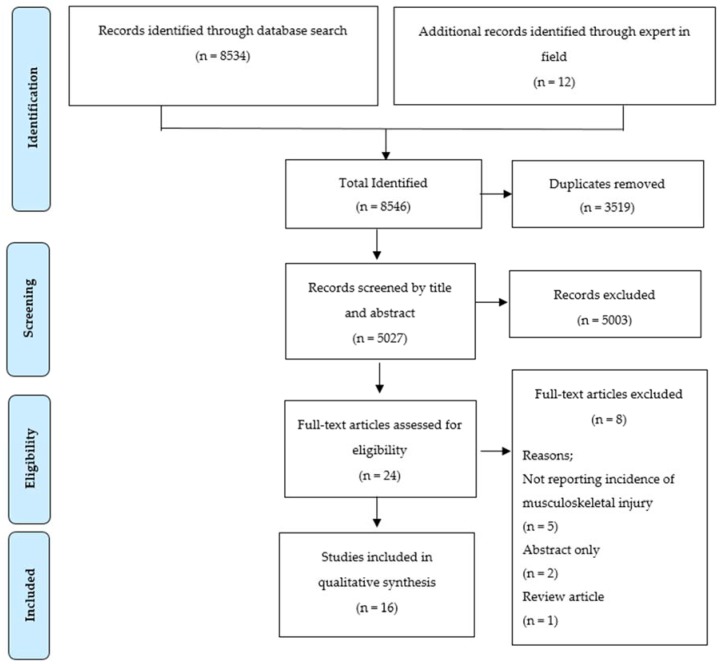
PRISMA flow diagram showing literature search, screening and eligibility results.

Following critical appraisal of the included studies, key data were extracted and tabulated from all included studies by two authors (K.L., C.R.) independently and then compared, with any discrepancies resolved by discussion and consensus to arrive at the final tabulated data set. Data extracted and tabulated for each study included author/s, year of publication, title, aim, study design, level of evidence, injury definitions, participant details and main findings of relevance to the aims of this review. The level of evidence provided by each included study was graded using published Australian National Health and Medical Research Council (NHMRC) criteria [32]. These criteria range from Level I (systematic review of all randomized control trials, highest level of evidence available) to Level IV (evidence obtained from case series, less reliable level of evidence [32]. For studies that were unable to be graded using these criteria, a note was made in the relevant data table. The PRISMA (Preferred Reporting Items for Systematic Reviews and Meta-analyses) guidelines were also used to guide this review (see Figure 1) and the reporting of included studies [33].

A critical narrative synthesis of key findings from the included studies was then conducted, with reference to the aims of the review, the tabulated data and further information provided in the original studies. In the synthesis, the findings from each included study were considered in the light of the respective study’s methodological quality, represented by its CAS and Kennelly [31] quality rating. Injury incidence rates were calculated or transformed to be represented as number of injuries per 1000 personnel per year, based on the available data from each of the included studies, provided the required data were reported. This allowed for comparisons of the reported injury incidence rates between the studies included in this review and between studies reported in this review and studies of other tactical populations (e.g., fire fighters, military). Due to the variations across studies in reporting methods and classification systems for the sites, natures and mechanisms of injuries, reported categories for each of these injury data elements were re-coded into broader groups, guided by previous studies in tactical populations [34], in order to allow for valid comparisons.

## 3. Results

The database search results are reported in Table 3. 

The PRISMA flow diagram (Figure 1) provides an overview of the results of the literature search, screening and selection processes. In total the initial search identified 8534 publications with an additional 12 publications sourced through experts in the field. Following removal of duplicates, 5027 studies were reviewed by title and abstract for potential eligibility, resulting in 24 full text publications being retrieved and evaluated in detail for eligibility against the inclusion and exclusion criteria. In total, 16 publications were deemed eligible and retained to form the basis for this critical narrative review.

The results of the critical appraisals of the methodological quality of the included studies [31], using the modified Downs and Black checklist [24], are shown in Table A1 as raw scores. Questions from the checklist that were ignored as planned due to lack of relevance to the research designs of the included studies are left blank. The final percentage CAS indicating the methodological quality of each study is listed in Table 4, along with information regarding the study’s aims, data sources, research designs, and the levels of evidence it provides. The Cohen’s kappa analysis revealed an ‘almost perfect agreement’ between raters (k = 0.977) [30] in the scores awarded to each study. One study was graded as being of ‘good’ methodological quality [15], seven studies were graded as being of ‘fair’ methodological quality [7,14,16,19,20,21,26], and eight studies were graded as being of ‘poor’ methodological quality [5,6,7,8,9,10,11,12,13,14,15,16,17,18,22,23,24,25]. The mean (±SD, Standard Deviation) percentage score for methodological quality of the included studies was 41.25% (±10.08%), with a range of 25.00% [6,14] to 65.00% [15].

Common weaknesses were found across all included studies (see Table A1) in the areas of reporting (three of eight questions), external validity (one of two questions), internal validity—bias (two of four questions), and internal validity—confounding/selection bias (two of five questions). As noted in Table 4, of the 16 studies included in this review only two [12,22] were determined to constitute level II evidence, due to their use of a prospective cohort study design. The remaining 14 studies [4,5,6,7,11,13,14,15,16,17,18,19,20,21] were cross-sectional or retrospective cohort studies and were therefore deemed to constitute levels of evidence [32] ranging between III-2 and IV.

Of the included studies, 10 were conducted in the United States [4,5,7,12,17,18,19,20,21,22], two in the United Kingdom [15,16], two in the Republic of Korea [13,14] and one in each of the Islamic Republic of Iran [11] and Australia [6]. Table 5 provides details of each study’s injury definition, participants and main findings.

**Figure 2:**
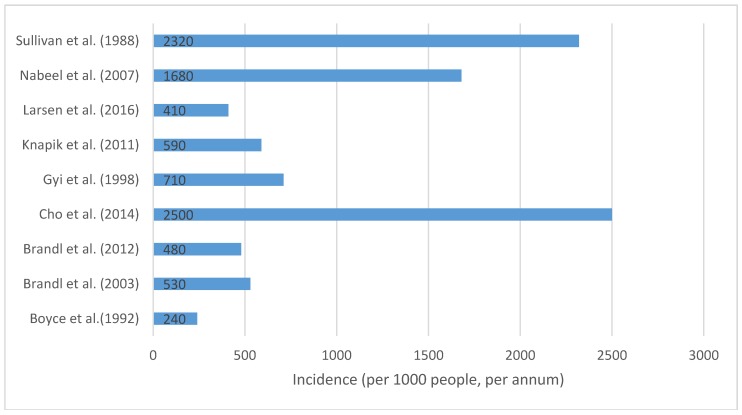
Injury incidence rates, by study (injuries per 1000 personnel per annum). Injury incidence rates are reported here only for studies which provided the required data to make these rates calculable. Studies not included in this figure did not supply adequate data for these calculations.

Injuries associated with different law enforcement occupational tasks were reported by seven of the studies [5,6,12,18,19,21,22]. There were varying ways that researchers gathered their injury data (Table 4). Five studies [4,5,18,21,22] collected data from workers’ compensation claims or reports, and three [13,14,19] collected their data from facilities to which law enforcement personnel presented with their injuries, e.g., hospitals. The remaining studies [7,11,12,20] gathered their data from law enforcement injury records filed by individual police departments or from surveys given to law enforcement personnel. For the one study [19] which collected data on emergency services personnel, only law enforcement officer data was extracted and used in this review. The observed injury incidence rates were calculable using data from nine of the included studies [4,6,12,13,15,18,20,21,22] (Figure 2). The reported incidence of injury for law enforcement personnel included in the studies reviewed varied from 240 [4] to 2500 [13] per 1000 personnel per annum, with no clear patterns evident based on particular population types or other contextual factors, for example injury reporting processes. Of the 16 included studies, only five [6,11,12,13,14] provided a clear definition of injury, with most only reporting the type of injury they were investigating, without providing a more comprehensive definition of exactly what comprised those types. This lack of clear injury definitions in most included studies prevented valid comparison of injury rates between studies and between the varying populations and contexts associated with the studies.

Five of the included studies [5,6,13,19,22] reported the body sites affected by recorded injuries. Of these, four studies [5,6,13,19] found that the most common site of injury was the upper extremity, with reported proportions of injuries that affected the upper limb ranging from 32.95% [5] to 43.42% [13]. The remaining study [22] reported the body site affected by 63.41% of injuries as ‘other (site not specified)’, indicating a lack of available detail in the data source, and also reported that a further 20.49% of injuries recorded in that study affected the back and torso. All five studies [6,12,18,19,21] that investigated the natures of the reported injuries agreed that sprains and strains were the most common nature of injury, with proportions of injuries these represented ranging from 42.36% [18] to 94.59% [6]. Of the seven studies [5,6,12,18,19,21,22] that examined how recorded injuries occurred, four [6,18,19,21] reported non-compliant offenders and associated assaults to be the most common cause, accounting for 31.5% [6] to 61.67% [21] of recorded injuries. Other, unspecified causes of injury, accounting for between 30% [22] and 41.7% [5] of recorded injuries, were reported by two studies [5,22], with the remaining study [12] reporting that 64.16% of recorded injuries occurred as a result of operational training.

There were only four studies [12,16,17,20] that discussed risk factors for injury within their results. One study [17] reported a link between increased body fat percentage and increased injury risk but did not provide any statistical data to support this. Another study [20] found that having a body mass index (BMI) greater than 35 kg/m^2^ tripled the likelihood of an officer having had back pain in the last 12 months, when compared to those with a BMI less than 35 kg/m^2^. Conversely, a third study [12] reported that physical characteristics such as BMI were poorly correlated with injury risk, but did not report any statistical data to support this assertion. A single study [16] investigated the association between lower back conditions and use of body armor, and found that those who wore body armor had a 9% higher rate of absenteeism due to lower back concerns than those who did not wear the body armor, though the statistical significance of this finding was not reported.

## 4. Discussion

The aim of this review was to identify and critically appraise recently published studies investigating musculoskeletal injuries in the law enforcement profession and then synthesise and report their findings. Many of the included studies not only investigated musculoskeletal injuries but other injuries as well, in these cases the musculoskeletal injury data were extracted and used within this review. With this in mind, it should be stated up front that, overall, the methodological quality of the eligible studies was only ‘poor’, with a mean (±SD) CAS of 41.25% (±10.08%). This relatively low score is in large part due to the majority of the study designs employed being susceptible to bias [35,36]. In particular, cross-sectional studies, including studies involving data collection at a single point in time using questionnaires, and retrospective study designs can introduce recall bias and difficulty in collecting complete data sets, and therefore often report lower injury incidence rates [23,36,37]. Thus, the findings of this review in relation to injury incidence rates are likely to be conservative and actual injury incidence rates are likely to be higher.

The main findings of this critical review formed five main categories of results for discussion. These were: (1) musculoskeletal injury incidence in law enforcement populations; (2) commonly injured body sites; (3) common natures of injury; (4) common mechanisms of injury (MOI); and (5) factors that may increase the risk of injury.

### 4.1. Musculoskeletal Injury Incidence

The reported incidence of injuries among law enforcement personnel varied from 240 [4] to 2500 [13] per 1000 personnel per annum, across the included studies. Variance in the reported injury incidence rates between studies could in many instances be attributable to research design factors like the included injury types, natures of the studies being compared, sources of data and injury definitions employed [38]. They could also be due to actual differences in injury rates, associated with differences in exposure to occupational tasks and risks and to differences in the levels of cohort resistance to injury, the latter resulting for example from enhanced levels of physical conditioning, from use of personal protective equipment, from development of specialized skill sets or from use of particular coercive tools, including weapons, to subdue offenders. Of the studies which reported injury incidence rates, the only study [4] to gather its data at the point of health care reported the lowest incidence rate. The three studies [13,20,22] with injury incidence rates that exceeded 1000 injuries per 1000 personnel per annum collected their injury data via surveys and workers’ compensation claims. Two of these three studies [13,20] collected all of their data via self-report, retrospectively, which relied on the officers’ recall ability and their knowledge of their previous injuries. This approach may not be as accurate as data collected at point of care or from a database which stores medical reports [37]. Finally, none of these three studies reported whether the total number of injuries included only the primary injury, or if secondary injuries were also reported and included, and inclusion and separate counting of secondary injuries incurred at the same time as the primary injuries may have artificially elevated reported injury incidence rates. 

Some research has already been completed looking at incidence of injuries amongst other tactical populations (e.g., military, fire fighters) [39,40,41]. Within the Australian army, incidence rates have been investigated in both full-time and part-time personnel [39]. The incidence rates were reported to be 169.3 and 301.9 per 1000 personnel per year, for full-time and part-time personnel respectively. Though these injury incidence rates are on the lower end of the findings of this review, musculoskeletal under-reporting of injuries is common in military populations, with observed injury under-reporting rates of up to 49% [42], and so these reported military injury incidence rates may be conservative for that population. In an Australian fire fighter population, retrospective data covering a nine year period was analyzed to determine an injury profile for this population [41]. Injury incidence rates from that study were reported to average 177 injuries per 1000 full-time employees per annum, which is a lower incidence rate than the rates reported in any of the studies included in this review, for law enforcement personnel. Similarly another study completed on firefighters in the United States also found the average annual incidence rate to be 177 (range of 136–215) injuries per 1000 personnel per annum [40]. That study was a descriptive study which analyzed data from fire department injury reports and annual summaries derived from injury surveillance databases. The observed differences in reported injury incidence rates among different tactical populations may be attributed to varying injury definitions, differences in data collection procedures and databases, and differences between the populations and their risk profiles and capacities to resist injury, as discussed above to explain differences between studies included in the current review. The varying natures of occupational tasks within different tactical fields (law enforcement vs. military vs. fire fighter) may also contribute to observed differences in reported injury incidence rates.

### 4.2. Affected/Injured Body Site

The most common body sites affected by injuries in law enforcement personnel were also reported differently across the included studies. For example, some studies reported ‘wrist/hand’ and ‘shoulder’ [6,13,19] while others [5,22] listed the site of injury more generally as ‘upper extremity’. When considered in broader categories, the most common sites of injury were the upper extremity, ‘other unspecified sites’, and the torso or back. Of the five included studies that investigated the reported sites of injuries, four [5,6,13,19] reported the upper extremity to be the most common site, representing 32.95% [5] to 43.42% [13] of all reported musculoskeletal injuries. The remaining study [22] reported ‘other sites’ to be the most common site of injury (63.41%). In this study, ‘other sites’ was not defined and it is therefore difficult to determine whether some of these injuries were perhaps not musculoskeletal injuries at all.

Other tactical populations, such as the military, have reported the lower limb and back to be the most common sites of injury [43,44,45,46,47,48]. In fire fighters, the most common sites of injury have similarly been reported to be the lower limb and the back rather than the upper extremity [40,41]. Differences in the most common sites of injury among tactical populations may be attributed to differences in injury definitions and in ways sites are reported and classified, and to how injury data was collected and the nature and focus of the study that was completed. In addition, a further notable contribution to site variations across tactical populations may be differences in occupational tasks, with load carriage requirements serving as an example, since different tactical populations carry different loads weights and configurations in different contexts. 

### 4.3. Nature of Injury

This review found the most common natures of injury to be reported by law enforcement personnel were sprains and strains, followed by other muscle pains and other natures of injury. All of the included studies that reported the natures of injuries sustained [6,12,18,19,21] found that sprains and strains were the leading natures of injury, however the reported percentages of all injuries that these natures accounted for varied from 42.36% [18] to 94.59% [6]. Unfortunately, the nature of injuries listed as ‘other muscle pain’ or ‘other’ was not further elucidated in the included studies. The wide variance across the included studies in the percentages of injuries with particular natures may again be explained by differences in study designs, methodological quality and data collection methods, as well as by risk profiles of each population and factors that counter those risks.

There have been similar findings in other tactical populations. In the military context, similar findings have been reported, with ankle and knee sprains representing 51% of reported injuries in one epidemiological study [49] completed on lower limb injuries occurring within New Zealand Defence Force personnel. Other studies have reported sprains and strains to be the most common natures of injury within military populations but have failed to report proportions of injuries these natures of injury represent [38,46]. Fire-fighting has been associated with a similar pattern, with the proportions of injuries that are sprains, strains and dislocations reported to be 40.2%–85.2% [40]. However, in this same population, other reported natures of injuries have included those of a non-musculoskeletal nature (e.g., thermal stress/heat exhaustion, chemical exposure, etc. [40,41,50]). 

### 4.4. Mechanism of Injury

The most common mechanism of injury reported by four of the seven included studies which examined mechanisms of injury in law enforcement personnel [6,18,19,21] was non-compliant offenders/assault, with percentages of injuries reported to arise from this mechanism varying from 31.5% [6] to 61.67% [21]. ‘Other unspecified causes’ were reported to be the most common mechanism of injury by a further two of the seven included studies [5,22] that examined mechanisms of injury, with reported percentages of injuries attributable to this mechanism in the range of 30% [22] to 41.71% [5]. Operational training was reported to be the most common mechanism of injury the final included study [12] that examined mechanisms of injury, associated with 64.16% of all reported injuries in that study. Like the other characteristics of injuries discussed so far (i.e., site and nature of injuries), the reporting of mechanisms of injury varied between studies. The differences between studies may be due to differences in methodological quality, study design and study aim, as well as to the way in which the data was collected and coded.

In contrast to the studies of law enforcement personnel, within the Australian Regular Army, 31.6% of injuries were attributed to the muscular stress associated with lifting, carrying or donning of equipment. The two most commonly reported activities within which injury occurred for these army personnel were physical training and operational training [51]. In fire fighters, it was found that no single mechanism of injury could explain more than 20% of injuries, and the most commonly reported mechanism was muscular stress, which accounted for 15.6% of reported injuries [41]. Differences in mechanisms of injury between tactical populations may be explained by differences in occupational demands. For example, military personnel are more likely to be carrying notably heavier loads [43], fire fighters are more likely to encounter hazardous environments [41], and law enforcement officers may be more likely to encounter non-compliant offenders [18,21]. Other than the differences in occupational demands, observed variations in reported mechanisms of injury across tactical fields may also be due to the study designs and methodological quality, or perhaps how data was collected (e.g., point-of-care versus self-reported methods of data collection).

### 4.5. Risk Factors for Injury

Potential risk factors for injury in law enforcement personnel were identified in this review but, as outlined in results, the findings were inconclusive. A few studies [12,17,20] investigated the link between increased BMI or increased body fat percentage and injury risk, with conflicting findings. One study [17] found a link between increased body fat percentage and increased injury risk, while another study [20] found that a BMI of more than 35 kg/m^2^ increased the likelihood of an officer having had back pain. Conversely, a third study [12] reported that physical characteristics such as BMI had little correlation with injury risk. One study [16] also looked at whether wearing body armor had an impact on lower back problems and found there was a correlation between days off due to back problems and wearing body armor. In a government report completed in the United States it was found that the proportion of law enforcement officers wearing bullet proof vests or body armor has been increasing [52]. As such, as more officers opt to wear body armor, the risk of back injury can be anticipated to increase.

In the military, there have been associations found between higher injury frequency and increasing age, hypo or hyper mobility, lower fitness levels and previous ankle sprains [46]. No correlation was found between increased body fat percentage or BMI and injury risk in one study [46], while another reported a 47% increase in musculoskeletal injury risk in army personnel who were overweight or obese [53]. In a fire-fighting population, a BMI of more than 30 kg/m^2^ has been shown to increase risk of musculoskeletal injury by 5.2 times [54]. Conversely, another study in this population [50] reported that body composition had no correlation with injury risk. As within law enforcement populations, there is mixed evidence for military and fire-fighting populations relating to whether body composition affects injury risk.

### 4.6. Limitations and Future Research

There were four key limitations in this review: (1) the modal methodological quality of included studies was only ‘fair’ and there was wide variance in the study designs across the included studies. Due to the methodological quality of the studies included in this review it is difficult to draw conclusive findings based upon their results. The majority of the included studies were retrospective in design and were either cross-sectional or cohort studies, and this combination of design features meant that there was an increased risk of bias [35,36] associated with the study findings; (2) each study reported different variables and the way in which they reported these varied; and due to differences in the injury classifications used across the included studies, broader categories had to be created to be enable valid comparison of results and as such some sensitivity was lost; (3) in the reported results, often there was no description or explanation of what the commonly-used ‘other’ category comprised; (4) the majority of the included studies had a male only sample, those which did include females were of a relatively low sample size. This limits the ability to be able to provide a gender analysis, and as such future research to investigate and profile gender specific injury may be of benefit to the volume of evidence available. 

Future research in this area needs to be conducted with better methodological quality. Larger, prospective cohort studies are needed, so that the results can be applied across the target population. Future studies should adopt a common injury definition and classification system, both of which remain to be developed for tactical populations, so that their findings can be compared with findings from other studies.

## 5. Conclusions

In conclusion, this review found that the current research literature surrounding musculoskeletal injuries affecting law enforcement personnel is limited in volume and of poor methodological quality. The reported incidence of injuries for law enforcement personnel varied from 240 to 2500 injuries per 1000 personnel per year and the most common site of injury was the upper extremity. Reported injuries were commonly sprains or strains caused by a non-compliant offender/assault. Caution is advised in extrapolating these findings to the wider law enforcement population due to the identified concerns regarding methodological quality and variability of reporting in the included studies.

## Figures and Tables

**Table 1:** Details of literature search: databases used and search terms (completed on 16 September 2016).

Database	Search Terms
PUBMED	(police OR law enforcement) AND (injury OR injuries)
CINAHL	(police OR law enforcement) AND (injury OR injuries)
EMBASE	(‘police’/exp OR police OR ‘law’/exp OR law AND enforcement) AND (‘injury’/exp OR injury OR injuries)
Ovid MEDLINE (®1946 to September Week 1 2016)	(police OR law enforcement) AND (injury OR injuries)

**Table 2:** Inclusion and exclusion criteria and examples of excluded studies.

**Inclusion Criteria**	**Example/s**
Study was focused on law enforcement officers	Studies involving police officers, a police department, or a police force
Study examined injuries occurring to, or in, a law enforcement population	Studies examining musculoskeletal injuries, musculoskeletal disability, injury epidemiology, injury rates, injury incidence
**Exclusion Criteria**	**Example/s**
Study involved participants who were not general Law Enforcement officers	Studies involving military police
Study examined injuries caused by police officers	Studies reporting police brutality, Taser injuries, excessive use of force by police
Study included only injuries that were not musculoskeletal injuries	Studies which only examined fatalities, chemical hazards, HIV, HEP B, Mortality, Homicide, Suicide, mental illness
Study reported as an abstract only	

**Table 3:** Databases and search results prior to screening and removal of duplicates.

Database	Identified Studies (*n*)
PUBMED	3845
CINAHL	634
EMBASE	1714
Ovid MEDLINE (®1946 to September Week 1 2016)	2341

**Table 4:** Key information regarding the design, aims, data sources, critical appraisal score and levels of evidence of each included study.

Authors (Year) and [Reference]	Title	Aim/Objective/Hypothesis	Study Design	Data Collection Method	Downs & Black Score	Level of Evidence *
Knapik et al. (2011) [12]	Injury rates and injury risk factors among federal bureau of investigation new agent trainees	To prospectively examine potential risk factors and injury rates in a cohort of FBI new agents.	Prospective Cohort Study	Database	65.00%	II
Violanti et al. (2013) [7]	Shift work and long-term injury among police officers	To determine whether the incidence of long-term injury leave varies across shifts.	Cross-sectional Study	Point of care	50.00%	IV
Cho et al. (2014) [13]	Factors Affecting the Musculoskeletal Symptoms of Korean Police Officers	To investigate efficient, systematic management of the Korean police and to examine the status and prevention of musculoskeletal disorders in Korean police officers.	Cross-sectional Questionnaire Study	Self-report	50.00%	IV
Boyce et al. (1992) [4]	Workers’ compensation claims and physical fitness capacity of police officers	To compare level of physical fitness with workers’ compensation claims among 514 police officers.	Cross-sectional Study	Point of care	50.00%	IV
Superko et al. (1988) [17]	Effects of a Mandatory Health Screening and Physical Maintenance Program for Law Enforcement Officers	To present findings regarding the effects of a mandatory health assessment, physical performance testing, and disease prevention program used by the California Highway Patrol.	Retrospective Cohort Study (Pre-Post Intervention Study using two retrospective cohorts)	Database	45.00%	IV
Gyi et al. (1998) [15]	Musculoskeletal problems and driving in police officers	To examine the effect of driving on sickness absence and prevalence data for musculoskeletal troubles of police officers.	Cross-sectional Questionnaire Study	Self-report	45.00%	IV
Burton et al. (1996) [16]	Occupational risk factors for the first-onset and subsequent course of low back trouble: a study of serving police officers	To determine the hazard for first-onset and subsequent course of low back trouble associated with occupational physical and psychosocial stressors.	Cross-sectional Questionnaire Study	Self-report	45.00%	IV
Sullivan et al. (1988) [22]	Epidemiological studies of work-related injuries among law enforcement personnel	To calculate the work-related injury only rates for employees working in different assignments; to see if there were sex or age differences in injury rates for workers performing similar assignments; to learn how the injuries occurred; and to examine the impact of these injuries in terms of days lost from work and claim costs.	(1) Cross-sectional descriptive study with ‘nested’/linked: (2) retrospective cohort study; and (3) prospective cohort study components	Database	45.00%	(1) IV (2) III-2 (3) II
Jahani et al. (2002) [11]	Musculoskeletal disabilities among police force personnel of the Islamic Republic of Iran	To assess the frequency of musculoskeletal disabilities in police force personnel and to determine the association between disabilities and age, rank, and different job types.	Retrospective Cohort Study	Database	40.00%	III-2
Holloway-Beth et al. (2016) [5]	Occupational Injury Surveillance Among Law Enforcement Officers Using Workers’ Compensation Data, Illinois 1980 to 2008	To use Illinois Workers’ Compensation Commission data to (1) determine the annual and cumulative claim rates for injuries suffered by law enforcement personnel; (2) describe the causes and nature of occupational injuries suffered by the four major groups of law enforcement officers; and (3) evaluate three important workers’ compensation outcomes related to long-term impacts of on-the-job injury or illness.	Retrospective Cohort Study	Database	40.00%	III-2
Nabeel et al. (2007) [20]	Correlation between physical activity, fitness, and musculoskeletal injuries in police officers	To explore whether Minneapolis police officers who had higher levels of fitness and physical activity has fewer musculoskeletal injuries than those who were not as active or fit.	Cross-sectional Questionnaire Study	Self-report	35.00%	IV
Reichard et al. (2010) [19]	Occupational injuries among emergency responders	To address the limited availability of nonfatal injury information for Emergency Medical Services personnel, fire fighters, and police (herein grouped as emergency responders) and the lack of comparable data, the authors analysed occupational injuries and illnesses among these workers which were treated in U.S. hospital emergency departments 2000–2001.	Retrospective Cohort Study	Database	35.00%	III-2
Brandl et al. (2012) [18]	The Physical Hazards of Police Work Revisited	To examine the extent to which injuries to police officers have changed from 1996–1998 to 2006–2008.	Retrospective Cohort Study	Database	35.00%	III-2
Brandl et al. (2003) [21]	Toward an Understanding of the Physical Hazards of Police Work	To analyze the nature and frequency of police injuries sustained either by accident or assault for police officers and comparing these to injuries sustained by fire fighters to develop a more complete appreciation of the relative hazards of police work.	Retrospective Cohort Study	Database	30.00%	III-2
Larsen et al. (2016) [6]	The Injury Profile of an Australian Specialist Policing Unit	To quantify the number of isolated versus multiple injuries in the force and to investigate the associated costs and time away from work following work-related injury.	Retrospective Cohort Study	Database	25.00%	III-2
Rhee et al. (2015) [14]	Prevalence of Musculoskeletal Disorders Among Korean Police Personnel	To investigate efficient, systematic management of the Korean police and to examine the status and prevention of musculoskeletal disorders in Korean police officers.	Retrospective Cohort Study	Database	25.00%	III-2

* NHMRC guidelines used to determine the level of evidence [32].

**Table 5:** Injury definitions, participants and main findings from each included study.

Authors (Year) and [Reference]	Title	Injury Definition	Participant Details	Main Findings
Knapik et al. (2011) [12]	Injury rates and injury risk factors among federal bureau of investigation new agent trainees	An injury case was defined as a new agent who sustained physical damage to the body and sought medical care or medical compensation one or more times during the Federal Bureau of Investigation (FBI) new agent training course.	FBI new agents (*n =* 531) Males (*n =* 426) 24.4–29.9 years (*n =* 188) 30.0–38.6 years (*n =* 238) Females (*n =* 105) 24.1–29.9 years (*n =* 60) 30.0–37.0 years (*n =* 45)	One or more injuries during FBI academy training was recorded for 35% of men and 42% of women. Defensive tactics training accounted for the highest number of total injuries at 58%. For both male and female injury risk was increased with slower 300 m sprint, 1.5 mile run, and lower points on the physical fitness test. In males, increased age was also a significant risk factor for injury. 590 injuries per 1000 personnel, per year.
Violanti et al. (2013) [7]	Shift work and long-term injury among police officers	No Injury definition	Police officers from the Buffalo Police Department (*n =* 419) Males (*n =* 312) Females (*n =* 107) Average age 43 years old	Night shift had the highest incidence of long-term injuries >90 days compared to afternoon and day shifts. Officers on night shift also had a greater risk of long-term injury compared to short-term. Incidence rate per 100,000 person hours: Day shift: 0.48 Afternoon shift: 0.59 Night shift: 1.33
Cho et al. (2014) [13]	Factors Affecting the Musculoskeletal Symptoms of Korean Police Officers	Definition of musculoskeletal disorders is damage to nerves/muscles in the neck, shoulder, waist, arm/leg, and surrounding body tissues caused by repetitive motion, inappropriate position, use of excessive force, physical contact with sharp surfaces, vibration, temperature, etc.	Police officers (*n =* 353) All male (100%) Mean age (Standard Deviation [SD]) = 52.92 (8.71) years old	Area of injury and prevalence: Shoulder (44.2%) Waist (41.4%) Neck/head (31.2%) Legs/foot (26.1%) Hand/wrist/finger (16.7%) Arm/elbow (14.7%) 2500 injuries, per 1000 personnel, per year.
Boyce et al. (1992) [4]	Workers’ compensation claims and physical fitness capacity of police officers	No Injury definition	Police officers (*n =* 514): Collected workers compensation (*n =* 124) Males (*n =* 96) Mean age (SD) = 33 (7.3) years old Females (*n =* 30) Mean age (SD) = 30.3 (5.3) years old Didn’t collect workers compensation (*n =* 390) Males (*n =* 340) Mean age (SD) = 36.3 (8.4) years old Females (*n =* 48) Mean age (SD) = 30.2 (5.9) years old	Significantly more females collected workers’ compensation payments than males (*p <* 0.002) Officers with the rank of sergeant or higher had significantly lower rates of claims for workers’ compensation (*p <* 0.001) Of the males that did collect workers compensation they were significantly younger (*p <* 0.001) The lowest workers compensation claims came from those who were either in the highest or lowest fitness level. 240 injuries, per 1000 personnel, per year.
Superko et al. (1988) [17]	Effects of a Mandatory Health Screening and Physical Maintenance Program for Law Enforcement Officers	No Injury definition	California Highway Patrol Officers Baseline testing (*n =* 4480) 98.2% male 1.8% female	Medical referrals and Job actions Significant decreases in annual sick days, job-related injuries and cost of injuries (*p <* 0.001) Body fat and Injuries Uninjured officers had a significantly lower percentage of body fat than those injured (*p <* 0.001) Injury rates not reported.
Gyi et al. (1998) [15]	Musculoskeletal problems and driving in police officers	No Injury definition	Study Group (*n* = 80) Traffic control officers Males (*n =* 79) Females (*n =* 1) Mean age (SD) = 37.65 (7.7) years old Control Group (*n =* 91) General duty officers Males (*n =* 87) Females (*n =* 4) Mean age (SD) = 36.76 (9.11) years old	Lower back was the most frequently reported area of discomfort for traffic control officers driving cars at 35% of all injuries. The mean prevalence of lower back conditions was 28% for the study and control groups. Over 12 months, traffic control officers had a higher incidence of >8 days missed due to lower back trouble when compared with the control. 710 injuries, per 1000 personnel, per year.
Burton et al. (1996) [16]	Occupational risk factors for the first-onset and subsequent course of low back trouble: a study of serving police officers	No Injury definition	Police officers (*n =* 1885) Royal Ulster Constabulary (exposed to heavy body armour) officers (*n =* 1508) Male (92%) Female (8%) Mean age (SD) = 38.3 (8.8) years old Greater Manchester Police officers (*n =* 377) Male (87%) Female (13%) Mean age (SD) = 37.5 (7.2) years old	Those who were exposed to either vibration or increased time wearing heavy body armour had an increased injury incidence rate. However no injury rates were reported.
Sullivan et al. (1988) [22]	Epidemiological studies of work-related injuries among law enforcement personnel	No Injury definition	Los Angeles County Sheriff’s Department Employees Descriptive Injury claims (*n =* 2167) RetrospectiveWorkers’ compensation claims (*n =* 417) Prospective Claims followed until close or up to 2 years (*n =* 261)	Descriptive No difference in injury rates between gender 30–34 age group showed the highest incidence of injury 27 injuries, per 100 employees, per year Retrospective Wrist/hand (*n =* 61), back (*n =* 42) and knee (*n =* 27) were the three most common areas of injury 38% of back injuries were repeat injuries Overexertion was significantly linked with new back injuries (*p <* 0.005) Prospective Back injuries caused the most days off full duty and cost more than all other injuries 2380 injuries, per 1000 personnel, per year
Jahani at al. (2002) [11]	Musculoskeletal disabilities among police force personnel of the Islamic Republic of Iran	Musculoskeletal disease cases were defined as any case of disabilities in which the diagnosis was in domain of musculoskeletal diseases, including skeletal/joint impairments or limitation of motion, muscle injuries, peripheral nerve neuritis or neuralgia in upper and lower extremities, neck and trunk.	Police force personnel musculoskeletal disability cases (*n =* 669) Mean age (SD) = 38.7 (7.5) years old	Musculoskeletal disorders accounted for 25.7% of all physical disability in the police force personnel. Back disorders accounted for 43.6% of total musculoskeletal disorders. Commissioned officers had the highest number of back disorders compared to non-commissioned officers (*p <* 0.0001) Non-commissioned officers had higher numbers of dislocations and fractures compared to commissioned (*p <* 0.001)
Holloway-Beth et al. (2016) [5]	Occupational Injury Surveillance Among Law Enforcement Officers Using Workers‘ Compensation Data, Illinois 1980 to 2008	No Injury definition	Law enforcement personnel claims (*n* = 18,892) 45% Correctional institutions Mean age (SD) = 37.5 (10) 26% Municipal police Mean age (SD) = 37.2 (8.9) 22% Sheriff’s department Mean age (SD) = 39.7 (10.3) 7% State police Mean age (SD) = 38.9 (8.9)	Upper extremity was the most common body part affected (>26% in all law enforcement) Correctional officers had the highest number of injuries, but the lowest severity
Nabeel et al. (2007) [20]	Correlation between physical activity, fitness, and musculoskeletal injuries in police officers	No Injury definition	Active duty police officers from the Minneapolis Police Department (*n =* 322) Patrol officers (*n =* 198) Administration (*n =* 63) Special assignment (*n =* 61) Mean age 38 years old	Body Mass Index (BMI) >35 kg/m^2^ = 3 times more likely to report back pain (95% Confidence Interval [CI] 1.17–9.66) Fair or poor rating of health = 8 times more likely to report chronic pain (95% CI 1.94–37.12) 1680 injuries per 1000 personnel, per year.
Reichard et al. (2010) [19]	Occupational injuries among emergency responders	No Injury definition	Emergency responders work-related injuries (*n =* 123,900) Of this 52% was Law enforcement officer work-related injuries (*n =* 64,800) Male (87%) Female (13%) <25 years old (*n =* 3000) 25–34 years old (*n =* 34,200) 35–44 years old (*n =* 18,100) >44 years old (*n =* 9500)	34% of law enforcement injuries were sprains/strains Law enforcement injuries, top 4 injury areas; 26% were to the leg and foot 19% were to the hand 17% were to the neck & back 17% were to the arm
Brandl et al. (2012) [18]	The Physical Hazards of Police Work Revisited	No Injury definition	Milwaukee Police Department officer 1996–1998 officers (*n =* 1713) Total injuries (*n =* 2867) 2006–2208 officers (*n* = 1604) Total injuries (*n =* 2112)	1996–1998 Sprain/strain (17.1%) Other muscle pain (17.2%) Occurred controlling/arresting subject (42.9%) 2006–2008 Sprain/strain (22.7%) Other muscle pain (20.2%) Occurred controlling/arresting subject (38.5%) 480 injuries, per 1000 personnel, per year.
Brandl et al. (2003) [21]	Toward an Understanding of the Physical Hazards of Police Work	No Injury definition	Patrol officers/detectives (*n =* 1700) Police injury reports (*n =* 1054)	Sprain/strain (18.9%) Other muscle pain (15.6%) Occurred controlling/arresting subject (43.8%) Medical attention sought (50.8%) Time off work (13.3%) Rate of injury incidents per officer = 0.610 530 injuries, per 1000 personnel, per year.
Larsen et al. (2016) [6]	The Injury Profile of an Australian Specialist Policing Unit	Injury was defined as any mild physical harm (e.g., bruises), or any major physical harm involving outpatient or inpatient treatment	Police officers (*n =* 170)	Injured officers (*n =* 138) Total injuries (*n =* 229) Injuries per employee (*n =* 2.57) Isolated injuries (34.9%) Multiple injuries (65.1%) Occurred during operational policing duty (48.9%) Attributed to non-compliant offender (31.4%) Sprain/Strain (61.1%) Top 3 injury areas; Hand/wrist (21%) Back (16.6%) Head/neck (16.2%) 410 injuries, per 1000 personnel, per year.
Rhee et al. (2015) [14]	Prevalence of Musculoskeletal Disorders Among Korean Police Personnel	Health disorders that occur in the neck, shoulder, waist, and upper and lower extremities, and adjacent tissues by hazardous factors such as repetitive motion.	Police officers who visited the Seoul police hospital 2009 (*n =* 40,963) 2010 (*n =* 48,184)	X-ray, Computed Tomography (CT) and MRI scans were most common for lower extremity and spine disorders (*p <* 0.05) Knee and lumbar examinations were the most frequent (*p <* 0.05)

BMI = Body Mass Index: CI = Confidence Interval: CT = Computed Tomography: FBI = Federal Bureau of Investigation: MRI = Magnetic resonance imaging: SD = Standard Deviation.

## Appendix A

**Table A1:** Downs and Black [24] Critical Appraisal results for all included studies.

Study (*n* = 16)	Scores Assigned, by Item Number from the Downs and Black Checklist *																											
	1	2	3	4	5	6	7	8	9	10	11	12	13	14	15	16	17	18	19	20	21	22	23	24	25	26	27	Raw Score
Knapik et al., (2011) [12]	1	1	1		0	1	1		1	1	1	1				1	0	1		1	0	0			0	1	0	13/20
Violanti et al., (2013) [7]	1	1	1		0	1	1		0	1	1	1				0	0	1		1	0	0			0	0	0	10/20
Cho et al., (2014) [13]	1	1	1		0	1	1		1	1	1	0				0	0	1		1	0	0			0	0	0	10/20
Boyce et al., (1992) [4]	1	1	1		0	1	1		0	1	1	1				0	0	1		1	0	0			0	0	0	10/20
Superko et al., (1988) [17]	1	1	0		0	1	1		0	0	1	0				0	1	1		1	1	0			0	0	0	9/20
Gyi et al., (1998) [15]	1	0	1		1	1	1		0	0	1	0				0	0	1		1	0	1			0	0	0	9/20
Burton et al., (1996) [16]	1	1	1		0	1	1		0	0	1	1				0	0	1		1	0	0			0	0	0	9/20
Sullivan et al., (1988) [22]	1	1	1		0	1	1		0	0	1	0				1	0	1		1	0	0			0	0	0	9/20
Jahani et al., (2002) [11]	1	1	1		0	1	1		0	0	1	0				0	0	1		1	0	0			0	0	0	8/20
Holloway-Beth et al., (2016) [5]	1	1	1		0	1	1		0	1	0	0				0	0	1		1	0	0			0	0	0	8/20
Nabeel et al., (2007) [20]	1	1	0		0	1	1		0	1	0	0				0	0	1		1	0	0			0	0	0	7/20
Reichard et al., (2010) [19]	1	1	0		0	1	1		0	0	1	0				0	0	1		1	0	0			0	0	0	7/20
Brandl et al., (2012) [18]	1	1	0		0	1	0		0	0	1	1				0	0	1		1	0	0			0	0	0	7/20
Brandl et al., (2003) [21]	1	1	0		0	1	0		0	0	1	1				0	0	0		1	0	0			0	0	0	6/20
Larsen et al., (2016) [6]	1	1	0		0	1	0		0	0	1	0				0	0	0		1	0	0			0	0	0	5/20
Rhee et al., (2015) [14]	1	1	0		0	1	0		0	0	1	0				0	0	0		1	0	0			0	0	0	5/20

* Questions from the checklist that were ignored as planned due to lack of relevance to the research designs of the included studies are left blank.
